# Safe Prostatic Artery Embolization with Cyanoacrylate: Representative Cases Demonstrating Non-Target Artery Preservation

**DOI:** 10.3390/diagnostics16121785

**Published:** 2026-06-10

**Authors:** Antonio Vizzuso, Antonio Spina, Maria Vittoria Bazzocchi, Emanuela Giampalma, Matteo Renzulli

**Affiliations:** 1 Radiology Unit, Morgagni-Pierantoni Hospital, AUSL (Azienda Unità Sanitaria Locale) Romagna, 47121 Forli, Italy; antonio.spina@auslromagna.it (A.S.); mariavittoria.bazzocchi@auslromagna.it (M.V.B.); emanuela.giampalma@unibo.it (E.G.); matteo.renzulli@unibo.it (M.R.); 2 Department of Medical and Surgical Sciences, University of Bologna, 40100 Bologna, Italy

**Keywords:** cyanoacrylate, N-butyl cyanoacrylate, prostatic artery embolization, benign prostatic hyperplasia, penile artery preservation, pelvic vascular anatomy, embolic control techniques, non-target embolization, atherosclerotic vascular disease

## Abstract

This study describes five case of prostatic artery embolization (PAE) using n-butyl cyanoacrylate methylsulfone (NBCA; Glubran 2, GEM; Viareggio, Italy) to evaluate its technical advantages, against standard particulate embolization, in managing complex pelvic vascular anatomy while preserving non-target arteries, with particular focus on penile artery protection. NBCA was mixed with iodized oil (Lipiodol Ultra Fluid; Guerbet, Aulnay-sous-Bois, France) at 1:5 dilution and injected in small aliquots (0.1–0.3 mL) under fluoroscopy using a blocked-flow technique, with injection stopped at reflux into the horizontal PA segment. After a few seconds, before the complete polymerization of the glue, the microcatheter is quickly withdrawn, flushed, and reused for the contralateral side. NBCA allowed precise embolic control through distinct mechanisms (direct distal, indirect distal, proximal, reflux and controlateral control), avoiding the need for protective coiling. Glue-based PAE seems a reproducible, safe, and efficient alternative to particulate embolization, offering enhanced embolization control and non-target artery preservation in anatomically complex patients.

**Figure 1:**
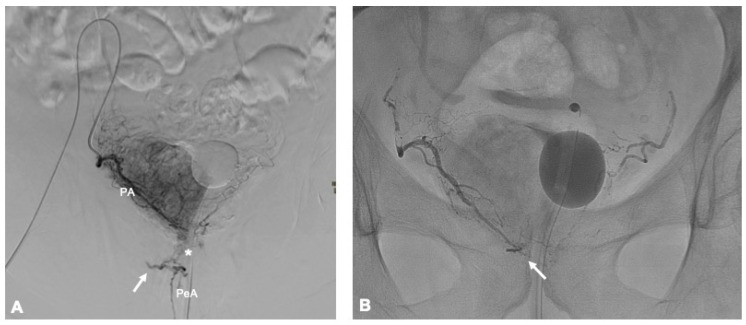
Case 1—Direct Distal Control. (**A**) Angiography of the right prostatic artery (PA) demonstrated retrograde opacification of the distal segment of the ipsilateral internal pudendal artery (IPA) (arrow) and anterograde filling of the right dorsal penile artery (PeA). This vascularization pattern was due to a dense anastomotic network of multiple small, non-catheterizable vessels located at the prostatic apex (asterisk). With the standard technique (using microspheres), the PA would have been navigated as distally as possible and a protective coil deployed downstream, followed by particle injection only after confirmation of dorsal PeA exclusion. Differently, in this case, the embolization was performed with NBCA. (**B**) The microcatheter was maintained in an extra-prostatic position, and glue was injected until the cast reached the distal portion of the right PA, with injection carefully interrupted just before crossing into the anastomotic capillary network at the apex (arrow). This approach, which we define as Direct Distal Control, ensured complete embolization of the prostatic supply while preserving PeA patency.

**Figure 2:**
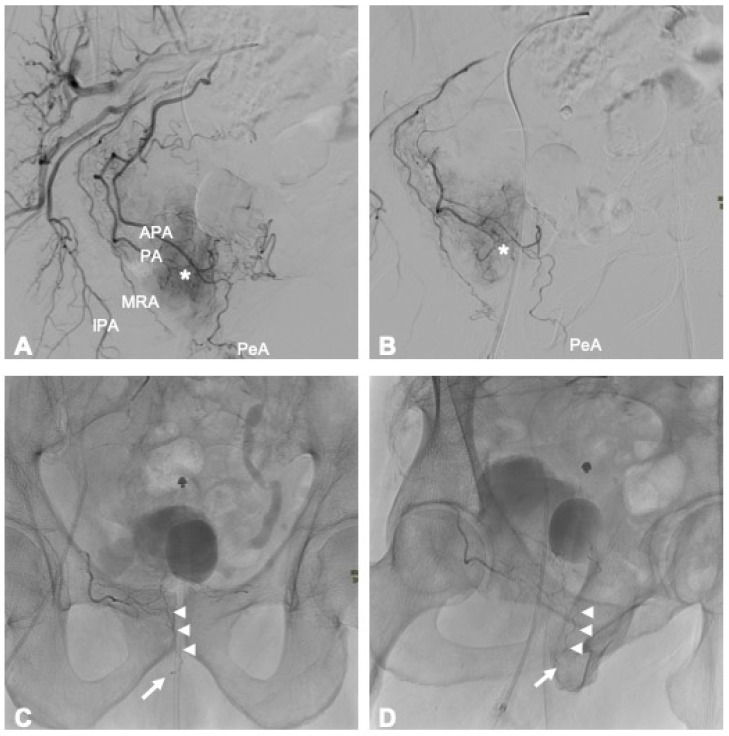
Case 2—Indirect Distal Control. (**A**) Right oblique projection of pelvic angiogram from the right hypogastric artery shows the PA arising from a common trunk with an accessory pudendal artery (APA), which coursed anteriorly and laterally toward the penile circulation, giving rise to the right dorsal PeA. The middle rectal artery (MRA) and internal pudendal artery (IPA) are also identified. Note the parenchymographic blush of the right prostatic lobe (asterisk). (**B**) Selective PA angiography demonstrated opacification of the right prostatic lobe (asterisk), but also of the distal segment of the APA and subsequently of the dorsal PeA, through a dense anastomotic network arising from the glandular parenchyma. With the standard technique (using microspheres), the APA would have required catheterization and coil deployment in its distal tract before proceeding with embolization. (**C**) Instead, NBCA embolization was performed directly from the PA, with the cast progressively advancing from the prostatic parenchyma into the distal segment of the APA (arrowheads). Injection was carefully interrupted before reaching the dorsal PeA (arrow). (**D**) Same acquisition as in panel C, with the C-arm rotated 45° to the right. Furthermore, the initial panoramic angiogram from the hypogastric artery had also shown additional supply to the right penile circulation from the ipsilateral IPA, thereby maintaining its patency.

**Figure 3:**
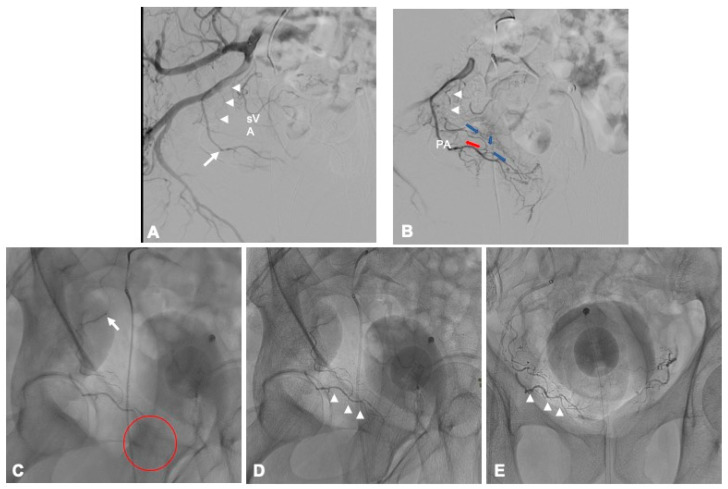
Case 3—Proximal Control. (**A**) Preliminary angiography of the right hypogastric artery demonstrated two PA (arrow and arrowheads) arising from a common trunk with the superior vesical artery (sVA). (**B**) After multiple attempts at selective catheterization, the more cranial and smaller PA (arrowheads) was catheterized; selective angiography showed that it did not supply the prostate directly but instead joined the middle segment of the other more representative PA. Distal advancement was not feasible. Because the catheter position was too proximal and distant from the prostatic parenchyma, particulate injection (standard technique using microspheres) would have resulted in unpredictable distribution due to progressive changes in peripheral resistance and flow dynamics. The alternative would have been to catheterize the main PA, a technically more demanding option often requiring additional time and effort in less favorable vascular anatomies. Conversely, NBCA allowed reliable control of embolic progression, as its behavior mirrored the distribution of the contrast agent. The images demonstrate both anterograde flow toward the gland (blue arrows) and retrograde flow (red arrow) along the PA, allowing prediction of liquid embolic distribution. (**C**) In this setting, embolization with NBCA was performed with the microcatheter tip positioned proximally (arrow). The glue cast extended distally into the right hemigland (red circle), achieving effective prostatic devascularization. (**D**) Oblique and (**E**) anteroposterior views show the distribution of the glue along the embolized vessels (arrowheads).

**Figure 4:**
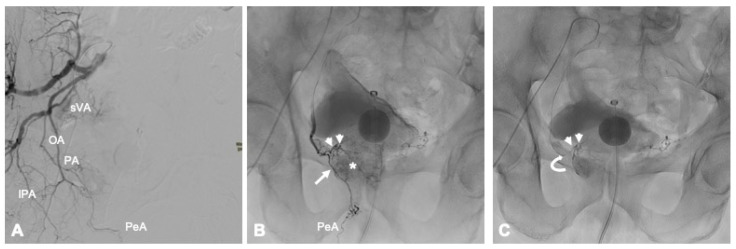
Case 4—Reflux Control. (**A**) Right oblique projection of the pelvic angiogram from the right hypogastric artery shows the sVA, obturator artery (OA), IPA, PA, and right dorsal PeA. (**B**) Angiogram of the right PA demonstrating a short prostatic branch (arrowhead) supplying the right prostatic lobe (asterisk) and a proximal collateral branch (arrow) directed toward the right PeA. In this setting, particulate embolization (standard technique [using microspheres]) would carry a high risk of reflux into this collateral; to avoid this scenario, coil occlusion could be considered. (**C**) Conversely, in this case, following selective catheterization of the short prostatic branch (arrowhead), embolization with NBCA was performed. The glue cast progressed until early reflux was observed at the microcatheter tip (curved arrow), at which point injection was stopped, just before the collateral to the dorsal PeA.

**Figure 5:**
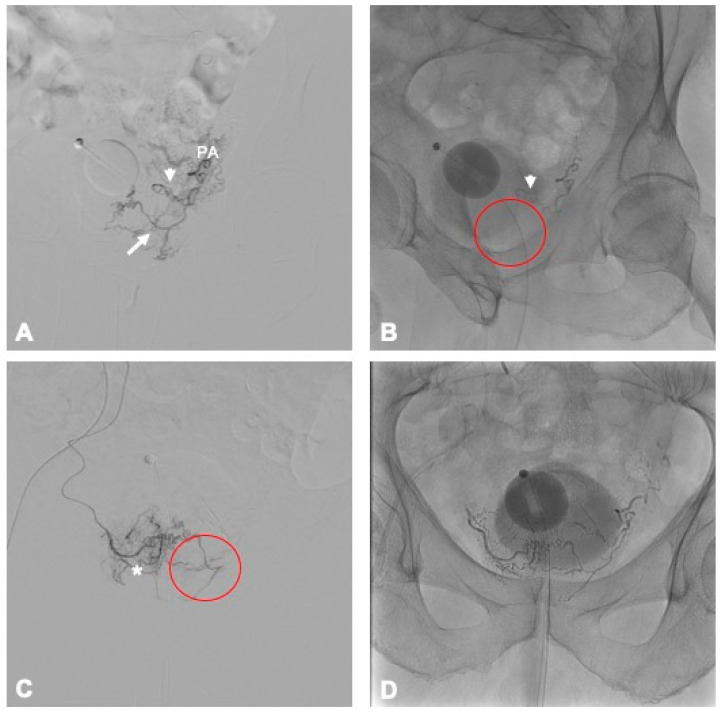
Case 5—Contralateral Control. (**A**) Angiography demonstrated the left PA bifurcated into a posterior–cranial branch supplying the median lobe (arrowhead) and an anterior–caudal branch (arrow). (**B**) After embolization with NBCA, the glue cast was visible in the posterior branch (arrowhead), while the anterior–caudal branch remained patent (red circle). (**C**) Anteroposterior angiogram of the right PA demonstrated perfusion of the right hemigland (asterisks) and the untreated portion of the left lobe (red circle), confirming cross-perfusion. (**D**) Anteroposterior images after embolization showed glue distribution across both prostatic lobes, providing the operator with confidence of complete gland embolization. All patients achieved sustained clinical improvement, with a reduction in IPSS (from 22–29 to 10–15) and prostate volume (mean reduction approximately 35%) without penile or other complications. Patients with indwelling catheters successfully resumed spontaneous voiding. Bilateral embolization was achieved in all cases, with no need for re-embolization. Follow-up was performed with magnetic resonance (MR) imaging over a period ranging from 24 to 36 months. The cyanoacrylate–lipiodol cast was partially reabsorbed on follow-up imaging, without causing artifacts that could interfere with the volumetric assessment of the gland. Demographic and clinical outcome data are reported in Supplementary Table S1. Performing PAE requires navigating one of the most intricate vascular districts of the body [1]. The PA shows significant variability in origin and may arise from multiple branches of the hypogastric circulation [1,2]. In addition, a complex collateral network, including connections with penile, vesical, rectal, and contralateral prostatic branches, further complicates embolization and increases the risk of non-target embolization [3,4,5]. This risk is particularly relevant in elderly patients, in whom atherosclerotic changes and vascular tortuosity make catheterization more challenging and favor the development of hazardous anastomoses [6]. Among these, connections with the penile circulation are of particular concern due to the potential for severe ischemic complications [7,8,9]. Furthermore, anatomical variability in penile arterial supply, including exclusive dependence on the IPA (62%), mixed (33%), or exclusive supply from an APA (5%), highlights the importance of careful evaluation to preserve adequate perfusion [10]. These factors are increasingly relevant given the rising age of patients undergoing PAE [11,12]. To address this complexity, pre-procedural imaging (computed tomography (CT) MR angiography) and intra-procedural guidance (Cone-Beam CT (CBCT)) are combined with superselective microcatheterization to ensure safe and distal embolic delivery [13]. When dangerous collaterals are identified, coil protection may be used to exclude them, while in other situations the procedure relies on extremely controlled injection under continuous fluoroscopic guidance [1]. Historically, the standard technique has been the slow-flow injection of calibrated microspheres (usually 300–500 μm), producing progressive ischemia of the gland [1,2]. This approach is well established and effective, but its limitations are also recognized: incomplete distal penetration, reopening of pre-existing anastomoses, potential recanalization of occluded branches, and the poor visualization of suspended particles in saline and contrast. These drawbacks may contribute to clinical recurrence and reduce operator confidence and perceived safety, particularly regarding the risk of non-target embolization [13,14,15,16]. Moreover, coil occlusion of non-target arteries requires advanced operator skill, increase in procedural costs, and prolonged acquisition times, with implications for radiation exposure and patient compliance, particularly in elderly patients. Furthermore, coil occlusion can only prevent embolization through macroscopically evident anastomoses, and thus fails to protect against embolization via anastomotic channels that form during the course of microsphere injection (slow embolization). Liquid embolic agents such as NBCA have gained increasing attention, offering clear advantages over particulates. NBCA ensures immediate and definitive occlusion, is clearly visible under fluoroscopy, and requires much smaller injection volumes, typically less than 1 mL per side versus 10–20 mL with microspheres. The rapid polymerization and viscosity of the NBCA–Lipiodol mixture not only shorten injection and fluoroscopy times but also minimize the risk of reflux and prevent the opening of anastomotic channels, thereby offering a potential safety advantage in anatomically complex cases. A high dilution (1:5) [11] was selected to achieve deeper distal penetration while maintaining adequate control of embolic progression when combined with the blocked-flow injection technique. With this technique, it is the operator who determines how far the glue progresses and when to stop, whereas particulate embolization, performed under free-flow injection, does not allow this level of control [11,12,13,14,17]. Particles are carried distally by blood flow, and in the presence of antegrade collaterals toward non-target territories, the operator cannot be certain that they remain confined within the prostatic parenchyma rather than reaching adjacent organs. The described technique, particularly in terms of dilution, may not be directly reproducible with other cyanoacrylate formulations, as differences in polymerization time and viscosity can significantly affect embolic behavior and require adjustment of injection parameters. Moreover, the use of glue may obviate the need for coils, further reducing procedural time and costs in complex cases. For instance, in Case 1, a coil could have been placed in the same PA that was subsequently embolized, whereas in Case 2, a different artery (APA) would have required catheterization and coiling, followed by re-catheterization of the PA, a technically demanding and undesirable step, especially given the difficulty of re-entering these vessels. Glue avoided this need, streamlining the procedure. These technical benefits make glue particularly suitable as a first-choice embolic agent in elderly patients with complex atherosclerotic pelvic vasculature [11]. Although comparative studies, as well as PAE-glue series, have not demonstrated a lower complication rate with glue compared with particles [11,12,13,14,17], it is important to note that, in order to achieve similarly low complication rates when using microspheres, all macroscopically visible anastomoses must be embolized. This approach entails higher procedural costs and inevitably longer fluoroscopy times, and requires a very high level of skill from interventional radiologists. All of these factors must be considered in a clinical context where the average age of patients is steadily increasing, and their compliance with lengthy procedures is progressively decreasing. PAE may also be considered within a multimodal therapeutic framework, and the enhanced embolic control achieved with NBCA may support future combined strategies, including surgical and pharmacological approaches, to improve symptom control in benign prostatic hyperplasia [18,19]. This study has limitations, including the small number of cases, its descriptive nature, and the absence of a control group to assess complications, as it is primarily intended to illustrate technical aspects rather than to provide definitive clinical conclusions. Although the choice of embolic material often depends on operator experience and preference, this series highlights that in this specific patient population the use of glue can offer clear technical advantages in terms of speed, practicality, and operator confidence. In particular, this case study challenges an outdated assumption that the use of glue is dangerous. On the contrary, the results suggest that the use of NBCA achieved technical success in complex vascular anatomies without penile complications.

## Data Availability

The original contributions presented in this study are included in the article/Supplementary Material. Further inquiries can be directed to the corresponding authors.

## Supplementary Materials

The following supporting information can be downloaded at: https://www.mdpi.com/article/10.3390/diagnostics16121785/s1, Table S1: Summary of clinical data.

## Abbreviations

The following abbreviations are used in this manuscript:

PAE	Prostatic artery embolization
NBCA	N-butyl cyanoacrylate
PeA	Penile artery
CT	Computed Tomography
MR	Magnetic Resonance
CBCT	Cone-beam CT
IPA	Internal pudendal artery
APA	Accessory pudendal artery
sVA	Superior vesical artery
OA	Obturator artery
