# Supplementary material for: Safe Prostatic Artery Embolization with Cyanoacrylate: Representative Cases Demonstrating Non-Target Artery Preservation
Source: Diagnostics (Basel). 2026 Jun 10;16(12):1785. doi: 10.3390/diagnostics16121785 (PMC13297664; doi:10.3390/diagnostics16121785)
Supplement: Supplementary file 1 [file diagnostics-16-01785-s001.zip › diagnostics-4251760-supplementary.pdf]

Table S1. Summary of clinical data

**Case**	Age (years)	Clinical Status	Baseline IPSS / QoL	Baseline PV (mL)	PAE Side	Post-PAE IPSS / QoL	Follow-up (months)	Post-PAE PV reduction (%)	Complications
**1**	82	Severe LUTS	29 / 5	95	Bilateral	11 / 2	36	39%	None
**2**	79	indwelling catheter	N/A	87	Bilateral	Catheter removed	24	29%	None
**3**	76	LUTS	27 / 6	120	Bilateral	15 / 2	36	40%	None
**4**	84	indwelling catheter	N/A	90	Bilateral	Catheter-removed	30	32%	None
**5**	76	LUTS	22 / 4	70	Bilateral	10 / 2	36	36%	None

Abbreviations: LUTS = Lower Urinary Tract Symptoms , IPSS = International Prostate Symptom Score, QoL = Quality of Life, PAE = Prostatic Artery Embolization, PV = Prostate Volume, N/A = Not Available
